# Effects of Understory or Overstory Removal on the Abundances of Soil Nematode Genera in a Eucalyptus Plantation

**DOI:** 10.3389/fpls.2021.640299

**Published:** 2021-06-01

**Authors:** Dandan Gao, Songze Wan, Shenglei Fu, Jie Zhao

**Affiliations:** ^1^Faculty of Life Science and Technology, Central South University of Forestry and Technology, Changsha, China; ^2^Key Laboratory of Agro-ecological Processes in Subtropical Region, Institute of Subtropical Agriculture, Chinese Academy of Sciences, Changsha, China; ^3^Collaborative Innovation Center of Jiangxi Typical Trees Cultivation and Utilization, College of forestry, Jiangxi Agriculture University, Nanchang, China; ^4^Key Laboratory of Geospatial Technology for the Middle and Lower Yellow River Regions, Ministry of Education, College of Environment and Planning, Henan University, Kaifeng, China; ^5^Huanjiang Observation and Research Station for Karst Ecosystems, Chinese Academy of Sciences, Huanjiang, China; ^6^National Engineering Laboratory for Applied Technology of Forestry and Ecology in South China, CSUFT, Changsha, China

**Keywords:** eucalyptus, soil nematode, life history strategy, principle response curve, vegetation removal

## Abstract

In south China, eucalyptus plantations typically consist of a single-species overstory (a eucalyptus monoculture) and a dense understory of a dominant fern species. In the current study, we assessed the effects of four treatments [control (CK), understory removal (UR), tree removal (TR), and all-plant removal (PR)] on the abundances of soil nematode genera, which can provide insight into the ecological functions of understory plants and trees. Soil nematodes were sampled six times (once before and five times after treatments were implemented) at 0–5 and 5–10 cm soil depths. The temporal dynamics of nematode genera were analyzed by the principle response curves (PRC) method. At 0–5 cm depth, the abundances of most nematode genera rapidly increased shortly after vegetation removal but then gradually decreased; the effects of UR were stronger than the effects of TR. The results might be explained by the pulsed input of plant debris to soil and its subsequent depletion. At 5–10 cm depth, the nematode communities were relatively unaffected by vegetation removal within the first 162 days, but the abundances of most genera sharply decreased on day 258 and then sharply increased on day 379 (the last sampling time). The results indicated that most nematode genera, even *r*-selected genera, were sensitive to vegetation removal in the upper soil layer and that understory vegetation can greatly affect soil nematode communities and presumably soil food webs. The nematode genera *Prismatolaimus* and *Diphtherophora* may be good indicators of the effects of vegetation removal. The results increase our understanding of the relationships between soil nematode genera and forest plant communities and of how soil biota is affected by forest management practices.

## Introduction

Soil nematodes are among the most abundant and diverse multicellular organisms in any environment on the planet (Bongers and Ferris, 1999; Coleman and Wall, 2015; Li et al., 2020). They occupy key positions in soil food webs and play important roles in maintaining soil biodiversity and in regulating biogeochemical cycling (e.g., carbon, nitrogen, and phosphorus cycles) (Neher, 2010; Zhang et al., 2019; Chen et al., 2020). Soil nematodes can therefore be useful for assessing soil food webs, soil ecological processes, and soil health (Ferris et al., 2001; Landi et al., 2018; Aira and Domínguez, 2020; Gao et al., 2021).

In most ecosystems, nematode communities are complex and consist of more than 30 taxa (Yeates and Bongers, 1999; van den Hoogen et al., 2019). Assessments of soil nematode communities have usually been based on functional group analysis (e.g., trophic groups, life history groups, or a combination of them) or have used community indices to condense community data into one variable in order to simplify analysis and interpretation (Neher and Darby, 2009; Cesarz et al., 2015; Li et al., 2015). A nematode taxon (e.g., genus) that represents a particular feeding type and life history strategy can theoretically be used as an ecological indicator or a variable for the analysis of ecosystem conditions (Yeates et al., 1993; Bongers and Bongers, 1998; Zhao and Neher, 2013), but nematode genera have rarely been used in this manner. The life history strategies of nematodes can be described on a colonizer–persister (cp) scale that ranges from 1 (*r*-selected taxa) to 5 (*K*-selected taxa). In addition, some taxa have key roles in controlling specific soil processes. For example, species of the nematode genus *Protorhabditis* mediate organic phosphate cycling in soil by feeding on *Mesorhizobium*, which is a keystone bacterial taxon that produces alkaline phosphomonoesterase (Jiang et al., 2017).

In most cases, however, the responses of nematode taxa to specific types of disturbance and therefore the ability of soil nematode to function as ecosystem indicators cannot be usefully assessed by analysis of variance and other traditional statistical techniques (Zhao and Neher, 2013), probably because the abundances of nematode taxa are highly variable in space and highly dynamic in time. Because of their ability to simultaneously analyze multiple variables and to visualize the data, multivariate statistical techniques, e.g., redundancy analysis (RDA) and canonical correspondence analysis (CCA), may be especially useful for assessing the responses of nematode taxa to disturbances or treatments (Neher and Darby, 2009; Zhao and Neher, 2013). For comparing the development of communities over time under changing conditions, the principle response curve (PRC), which is a more advanced multivariate statistical technique based on partial RDA, is useful (Lepš and Šmilauer, 2003). The current study concerns the use of the PRC method to study soil nematode taxa responses to plant removal in a eucalyptus plantation.

Because of their rapid growth and high productivity, eucalyptus trees have been widely planted and intensively managed for pulpwood production in many tropical and subtropical regions of the world (Chen et al., 2011). Although eucalyptus plantations are considered monocultures, in the subtropics they commonly contain understory vegetation that is often dominated by the fern *Dicranopteris* sp., which forms monospecific thickets (Zhao et al., 2012; Yang et al., 2021). Understory vegetation is thought to compete with target trees for soil water and nutrients and has traditionally been removed by farmers. However, previous studies have found that a *Dicranopteris*-dominated understory can greatly affect the soil microclimate, soil microorganisms, litter decomposition, nutrient cycling, tree-seedling regeneration, and stand productivity (Zhao et al., 2011; Wan et al., 2014). The effects of the overstory vegetation (i.e., the trees) on soil animals, in contrast, have seldom been studied. Research is also needed to determine how the temporal dynamics of soil animals are affected by forest management and how the characteristics of overstory and/or understory vegetation correlate with the abundance of soil animal taxa at a fine classification level.

The objective of this study was to use PRC analysis to determine how the temporal dynamics of soil nematode genera are affected by understory and/or overstory removal. To accomplish this objective, we used an existing dataset of the abundances of soil nematode genera in eucalyptus plantations. The data were collected as part of a study by Zhao et al. (2012), who assessed the soil nematode community on six dates in eucalyptus plantations subjected to four treatments, including control (CK), understory removal (UR), tree removal (TR), and all-plant removal (PR). The collected data on nematode trophic group abundances were published by Zhao et al. (2012), but the data on nematode genera have not been published. Since forest plants, including both overstory and understory, are the main food resources for soil biota (Wardle et al., 2004). In addition, they could maintain soil micro-climates (Zhao et al., 2011; Wan et al., 2014). In the current study, therefore, we tested two hypotheses: 1) both the understory and overstory removal would decrease the abundances of the nematode genera in upper and lower soil layers; and 2) the effects of vegetation removal on the abundances of nematode genera would increase over time.

## Materials and Methods

This study was conducted at the Heshan Hilly Land Interdisciplinary Experimental Station (112°50′E, 22°34′N), property of the Chinese Academy of Sciences (CAS) in Guangdong Province, China. The climate is subtropical monsoon with a distinct wet and dry season. The mean annual temperature and precipitation are 21.7°C and 1,700 mm, respectively. The soil is an acrisol (FAO, 2006). Vegetation at the experimental site consisted of three replicate 4-year-old *E. urophylla* plantations. Detailed information about the experimental design can be found in Zhao et al. (2012). In brief, four plots (15 × 15-m) were designated in each of the three replicate eucalyptus plantations and were randomly assigned to one of the following four treatments: CK, UR, TR, and PR. For the UR and PR treatments, the shoots of all understory plants were manually cut using a reaping hook. For the TR and PR treatments, the trees were cut at ground level using an electric saw. Most of the plant debris was immediately removed from the treated plots but the small pieces of debris (<2 cm) were not removed.

Soil samples were first collected on 20 September 2009 (T1), before the vegetation removal treatments were implemented on 30 September 2009. Soil samples were then collected on five subsequent dates (8 October and 6 November 2009; and 11 March, 15 June, and 14 October 2010; T2, T3, T4, T5, and T6, respectively). Soil cores (2.5 cm diameter) were taken at 0–5 cm and 5–10 cm depths from eight randomly selected locations in each plot and were combined to form one composite sample per plot. The surface litter was carefully removed before soil cores were taken. The samples were immediately transported to the laboratory in insulated boxes. Nematodes were immediately extracted from 50 g of moist soil *via* the Baermann funnel method for 48 h. The extracted nematodes were heat-killed and fixed in a 4% formalin solution. The nematodes were counted with an inverted microscope (Eclipse Ts100, Nikon). The first 100 individuals encountered were identified to genus with the aid of a differential interference contrast microscope (ECLIPSE 80i, Nikon) (Bongers, 1988; Jairajpuri and Ahmad, 1992; Mai and Mullin, 1996; De Ley et al., 2001). The identified nematodes were further assigned to trophic groups (bacterivores, fungivores, herbivores, omnivores, and carnivores) (Yeates et al., 1993; Okada et al., 2005) and colonizer–persister (cp) scales (Bongers and Bongers, 1998). The cp scale is a way of describing of nematode life history strategies with range from 1 (typical r-selected taxa) to 5 (typical K-selected taxa).

Repeated-measures ANOVA with SPSS 16.0 software (SPSS Inc., Chicago, IL, United States) was used to determine the effects of the treatments on the abundance of each nematode genus over time. The PRC method was used to determine the temporal dynamics of soil nematode generic composition as affected by vegetation removal treatments at 0–5 and 5–10 cm depths using CANOCO 4.5 (Ithaca, NY, United States). Data were log-transformed for PRC analysis to improve the quantitative interpretations of the PRC diagrams (Lepš and Šmilauer, 2003). The PRC only deals with two factors which are the treatment and the sampling time. Sampling time is used as a covariate. The control treatment was considered to be a zero baseline (the horizontal line). For the PRC analysis, the treatment effect is based on the temporal differences between control and experimental treatments. The result is a diagram showing the first principal component (RDA) of the variance explained by treatment on the Y-axis along the sampling dates on the X-axis, and a vertical graph on the right site shows the taxa scores. The deviation of abundance of a nematode genus from the control at each sampling event can be calculated with the following equation: exp(value in curve × value of genus score on the first RDA axis). The higher the score the more the actual response pattern of the nematode genus is likely to follow the pattern in the PRC. Nematode genera with high negative scores are inferred to show the opposite pattern, whereas nematode genera with near zero scores either show no response or a response that is unrelated to the pattern shown in PRC. The detailed procedure of PRC could be find in chapter 15.8 in Lepš and Šmilauer (2003). Monte Carlo permutation tests were used to compute statistical significance (*n* = 350).

## Results

**FIGURE 1:**
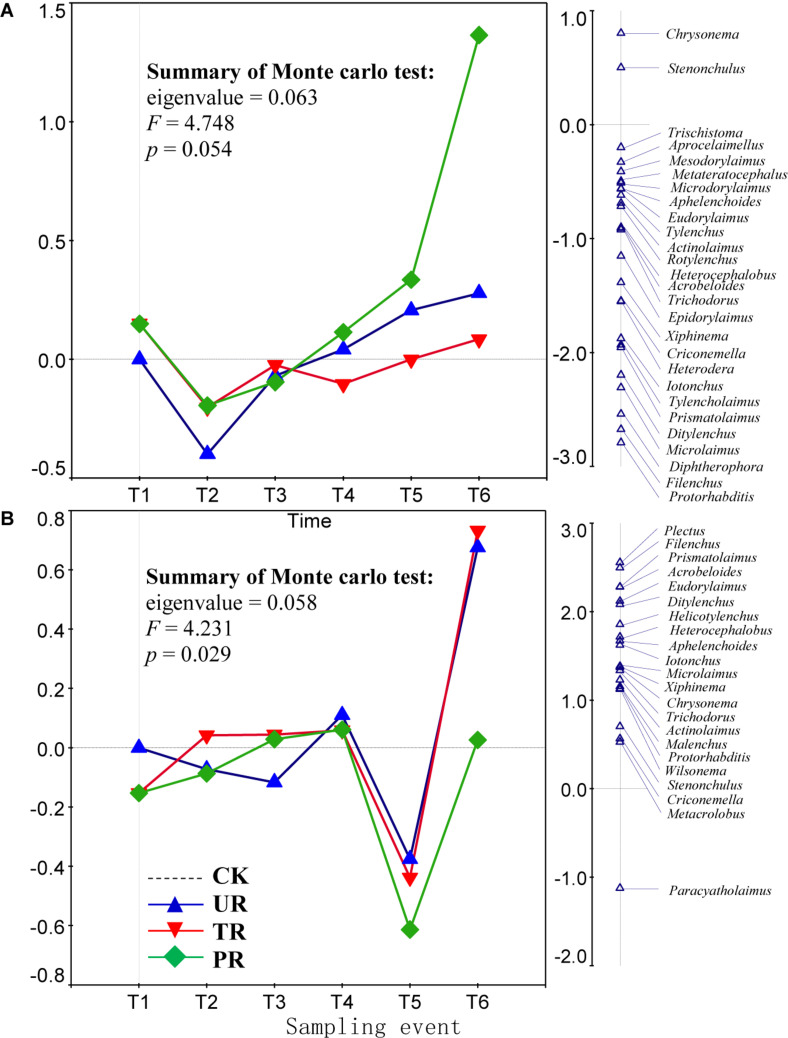
Principal response curves (PRC) with weights of abundance of nematode genera in control (CK), understory removal (UR), tree removal (TR), and all-plant removal (PR) plots at **(A)** 0–5 cm and **(B)** 5–10 cm soil depths across six sampling events. The bi-plot on the left side representing temporal trajectories of nematode community for each of the treatments (CK, UR, TR, and PR). The vertical graph on the right side showing the nematode genus scores. The horizontal dotted line represents the control treatment. At each sampling event, the greater the deviation of a response curve (treatment curve) from horizontal line, the greater the differences of nematode communities between the treatment and control. T1–T6 are 10 days before and 8, 37, 162, 258, and 379 days after vegetation removal treatments were applied, respectively. The nematode genera with scores close to 0 are not shown on the first RDA axis (the right side of the plot). The deviation of abundance of a nematode genus from the control at a sampling time can be calculated with the equation: exp(value in curve × value of genus score on the first RDA axis). Particularly, values > 1 indicate increase in the abundances of nematode genera relative to CK, and values < 1 indicate decreases in abundances of nematode genera relative to CK.

**FIGURE 2:**
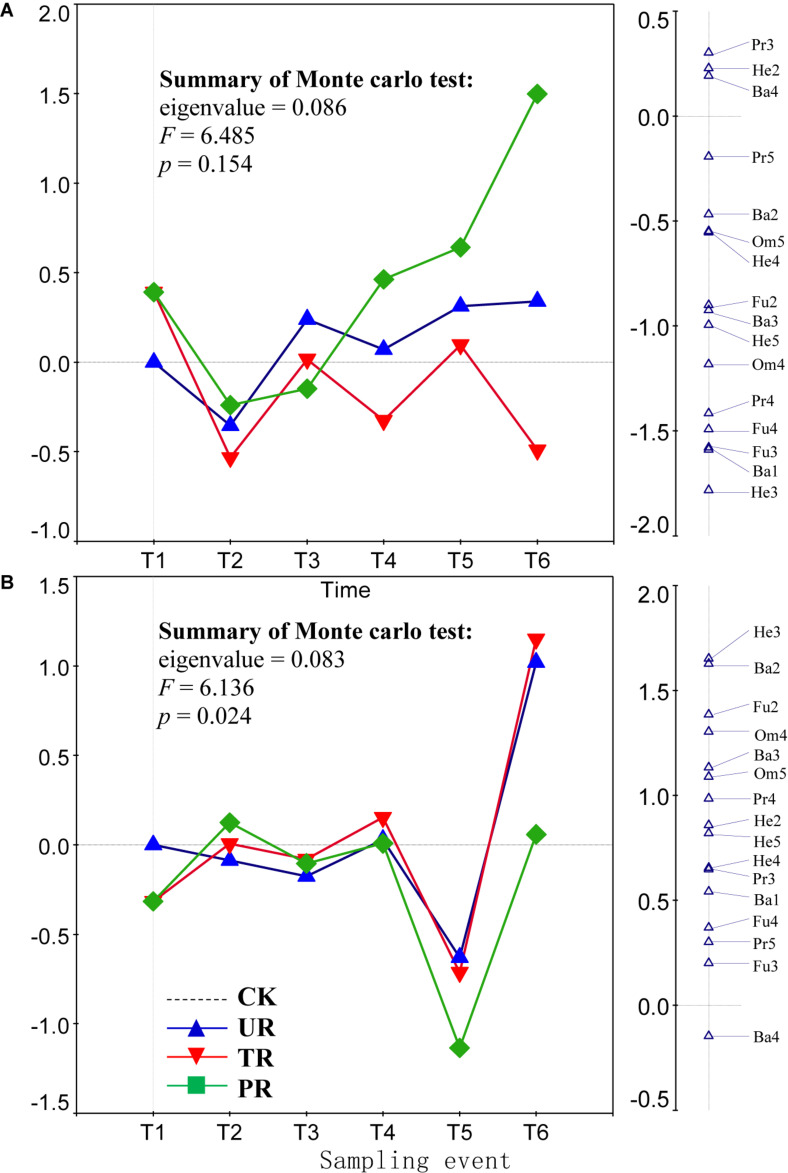
Principal response curves (PRC) with weights of abundance of nematode functional guilds in control (CK), understory removal (UR), tree removal (TR), and all plant removal (PR) plots at **(A)** 0–5 cm and **(B)** 5–10 cm soil depths across six sampling events. The bi-plot on the left side representing temporal trajectories of nematode community for each of the treatments (CK, UR, TR, and PR). The vertical graph on the right side showing the nematode functional guild scores. The horizontal dotted line represents the control treatment. At each sampling event, the greater the deviation of a response curve (treatment curve) from horizontal line, the greater the differences of nematode communities between the treatment and control. T1–T6 are 10 days before and 8, 37, 162, 258, and 379 days after vegetation removal treatments were applied, respectively. The deviation of abundance of a nematode functional guild from the control at a sampling time can be calculated with the equation: exp(value in curve × value of functional guild score on the first RDA axis). Particularly, values > 1 indicate increase in the abundances of nematode functional guilds relative to CK, and values < 1 indicate decreases in abundances of functional guilds relative to CK. Soil nematode functional guild designation is the composite of trophic group and cp value: Ba, bacterivore; Fu, fungivore; Pr, predator; Om, omnivore; He, herbivore.

A total of 62 nematode genera were collected (Supplementary Table 1). The most common genera were *Ditylenchus*, *Filenchus*, *Acrobeloides*, *Prismatolaimus*, *Aphelenchoides*, and *Protorhabditis*. Other common genera included *Eucephalobus*, *Helicotylenchus*, *Criconemella*, *Plectus*, and *Wilsonema*. Repeated-measure ANOVA did not find any significant effect of vegetation removal on each of the 62 nematode genera at 0–5 or 5–10 cm soil depth (Supplementary Table 1). The PRC method, in contrast, revealed that on the first sampling after plant removal (T2), vegetation removal increased the abundances of most nematode genera (44 of 58) at 0–5 cm soil depth (Figure 1A). At T2 and at 5 cm depth, the positive effect on the abundances of nematode genera was strongest for treatment UR and was equivalent for treatments PR and TR. At 5 cm depth and beginning at T3 and continuing until the end of the experiment (T6), the abundances of most nematode genera declined in response to all three vegetation-removal treatments (Figure 1A); the decline was greatest with PR, was lowest with TR, and was intermediate with UR, i.e., the effect of UR was greater than the effect of TR. At T6, the abundances of the common genera *Protorhabditis*, *Filenchus*, *Ditylenchus*, *Prismatolaimus*, *Criconemella*, *Acrobeloides*, and *Aphelenchoides* were lower in PR plots than in CK plots by about 98, 98, 95, 93, 88,72, and 54%, respectively; were lower in UR plots than in CK plots by about 58, 57, 50, 46, 38, 25, and 16%, respectively; and were lower in TR plots than in CK plots by about 20, 19, 16, 14, 12, 7, and 4%, respectively (Figure 1A and Table 1). In addition, nematode genera with high cp values (4 and 5) (e.g., *Tylencholaimus*, *Iotonchus*, *Epidorylaimus*, *Actinolaimus*, and *Eudorylaimus*) (Supplementary Table 1) were less sensitive to vegetation removal than the genera with low cp values (1 and 2) (e.g., *Protorhabditis*, *Filenchus*, and *Ditylenchus*) (Supplementary Table 1; and Figure 1A) and a cp5 genus (i.e., *Chrysonema*) was increased by vegetation removal (Figure 1A), which contrary to the nematode cp scale theory. The PRC with weights of nematode functional guilds at the 0–5 cm soil depth showed a similar pattern with that of nematode genera (Figure 2A). In addition, vegetation removal decreased most of the functional guilds except Pr3, He2, and Ba4 (Figure 2A).

**TABLE 1:** The relative deviation of nematode genera under vegetation removal treatments from the control at the fifth (T5) and sixth (T6) sampling events at the 0–5 cm and 5–10 cm soil depths.

Nematode genus	0–5 cm						5–10 cm					
	T5			T6			T5			T6		
	UR vs. CK ^a^	TR vs. CK	PR vs. CK	UR vs. CK	TR vs. CK	PR vs. CK	UR vs. CK	TR vs. CK	PR vs. CK	UR vs. CK	TR vs. CK	PR vs. CK
*Acrobeloides*	−17% ^b^	−1%	−27%	−25%	−7%	−72%	−58%	−63%	−76%	370%	435%	4%
*Actinolaimus*	−13%	−1%	−21%	−19%	−5%	−61%	−37%	−42%	−54%	131%	148%	2%
*Aphelenchoides*	−11%	−1%	−17%	−16%	−4%	−54%	−47%	−52%	−65%	210%	241%	3%
*Aporcelaimellus*	−7%	0%	−11%	−10%	−3%	−37%						
*Chrysonema*	18%	1%	31%	28%	7%	202%	−41%	−45%	−57%	154%	175%	2%
*Criconemella*	−27%	−2%	−41%	−38%	−12%	−88%	−19%	−22%	−30%	47%	52%	1%
*Diphtherophora*	−41%	−2%	−58%	−55%	−18%	−97%						
*Ditylenchus*	−37%	−2%	−53%	−50%	−16%	−95%	−55%	−60%	−73%	311%	362%	3%
*Epidorylaimus*	−21%	−1%	−32%	−30%	−9%	−80%						
*Eudorylaimus*	−11%	−1%	−17%	−16%	−4%	−54%	−55%	−61%	−73%	319%	373%	3%
*Filenchus*	−43%	−3%	−60%	−57%	−19%	−98%	−61%	−67%	−79%	442%	524%	4%
*Heterocephalobus*	−17%	−1%	−27%	−25%	−7%	−72%	−51%	−56%	−68%	252%	291%	3%
*Heterodera*	−27%	−2%	−41%	−38%	−12%	−88%	−47%	−53%	−65%	215%	247%	3%
*Iotonchus*	−32%	−2%	−47%	−44%	−14%	−93%	−46%	−51%	−64%	200%	229%	3%
*Malenchus*							−36%	−40%	−51%	119%	134%	2%
*Mesodorylaimus*	−8%	0%	−13%	−12%	−3%	−44%						
*Metacrolobus*							−18%	−21%	−28%	43%	47%	1%
*Metateratocephalus*	−10%	0%	−16%	−15%	−4%	−50%						
*Microdorylaimus*	−10%	0%	−16%	−15%	−4%	−50%						
*Microlaimus*	−38%	−2%	−55%	−51%	−17%	−96%	−41%	−46%	−58%	155%	176%	2%
*Paracytholaimus*							54%	65%	103%	−54%	−57%	−2%
*Plectus*							−62%	−67%	−79%	458%	544%	4%
*Prismatolaimus*	−33%	−2%	−49%	−46%	−14%	−93%	−58%	−63%	−76%	370%	435%	4%
*Protorhabditis*	−44%	−3%	−61%	−58%	−20%	−98%	−35%	−39%	−50%	114%	128%	2%
*Rotylenchus*	−14%	−1%	−22%	−20%	−6%	−63%						
*Stenonchulus*	11%	0%	19%	17%	4%	101%	−23%	−27%	−35%	61%	68%	1%
*Trichodorus*	−17%	−1%	−27%	−25%	−7%	−72%	−40%	−45%	−57%	149%	169%	2%
*Trischistoma*	−4%	0%	−7%	−6%	−2%	−25%						
*Tylencholaimus*	−33%	−2%	−48%	−45%	−14%	−93%						
*Tylenchus*	−12%	−1%	−19%	−18%	−5%	−58%						
*Wilsonema*							−35%	−39%	−50%	114%	128%	2%
*Xiphinema*	−25%	−1%	−38%	−35%	−10%	−85%	−41%	−46%	−58%	155%	176%	2%

*^*a*^The percentage is calculated with the following equation: [exp(value in curve × value of genus score on the first RDA axis) – 1] × 100%. The curve value and genus score are extracted for the PRC figure (i.e., Figure 1). ^*b*^Positive and negative values indicate the abundances of nematode genera of UR, TR, or PR are higher and lower than that of CK, respectively. CK, control; UR, understory removal; TR, tree removal; PR, all-plant removal.*

At the 5–10 cm soil depth, vegetation removal had only mild effects on the soil nematode communities at the first four sampling events (T1–T4) (Figure 1B). In the next two sampling dates, however, the abundances of most of the nematode genera (44 in 57) were first sharply decreased by plant removal (T5) and then sharply increased by plant removal (T6) relative to the CK treatment (Figure 1B). At T5, the abundances of the common genera *Plectus*, *Filenchus*, *Prismatolaimus*, *Acrobeloides*, *Ditylenchus*, *Helicotylenchus*, *Aphelenchoides*, *Protorabditis*, *Wilsonema*, and *Criconemella* were lower in PR plots than in CK plots by about 79, 78, 76, 76, 73, 68, 65, 50, 50, and 30%, respectively; were lower in UR plots than in CK plots by about 62, 61, 58, 58, 55, 51, 47, 35, 35, and 19, respectively; and were lower in TR plots than in CK plots by about 67, 66, 63, 63, 60, 56, 52, 39, 39, and 22%, respectively (Figure 1B and Table 1). At T6, the abundances of nematode genera in UR and TR plots were similar but were much higher than those in CK plots, and nematode abundances in PR plots were similar to those in CK plots (Figure 1B and Table 1). The PRC with weights of nematode functional guilds showed a similar pattern with that of nematode genera at the 5–10 cm soil depth (Figure 2B).

## Discussion

In this study, we used the PRC method to visualize the temporal dynamics of soil nematode genera as affected by removal of understory and/or overstory plants. We first determined that these responses to plant removal were not evident based on repeated-measures ANOVA. Soil nematode communities in most ecosystems consist of more than 30 genera (Yeates and Bongers, 1999). In this study, 62 genera were recorded, and in many cases their abundances had high levels of variance (Supplementary Table 1). Therefore, even the dominant nematode genera (e.g., *Ditylenchus*, *Filenchus*, and *Acrobeloides*) did not show significant responses to the vegetation removal treatments according to ANOVA. The failure of conventional statistical methods, e.g., ANOVA, to detect temporal changes in complex communities in response to multiple treatments is the most primary reason for using the PRC method (Van den Brink and Ter Braak, 1998; Neher and Darby, 2009).

Partially consistent with our first hypothesis, our PRC analysis revealed that vegetation removal in a eucalyptus plantation reduced the abundance of most soil nematode genera in the upper soil layer over time; however, the abundances were initially increased in response to vegetation removal. The early and short-term increase in the abundances of nematode genera in response to vegetation removal presumably resulted from a pulsed input of plant debris into soil. In consistent with this finding, previous studies reported that pulsed inputs of litter following clear-cut harvesting increased soil nematode abundance in pine forests in Sweden (Sohlenius, 1982, 1997). However, an increase in soil nematode abundance in response to pulsed litter inputs resulting from vegetation removal was reported seldomly, which may due to the studies focused on early and short-term effects (e.g., several days or weeks after) are scarce. In addition, the initial increases of nematode abundances may also result from the elimination of allelopathy effects after of eucalyptus and/or *Dicranopteris* sp. removal. The eucalyptus and dicranopteris are reported to be allelopathy species (Chong and Ismail, 2006; Zhang et al., 2016), which potentially affect soil biota too (Lankau, 2012). Regardless, there is a consensus that vegetation removal will decrease soil nematode abundances in the long-term due to a reduction in food resources for microorganisms and consequently for most nematodes (Sohlenius, 1996; Forge and Simard, 2000; Landi et al., 2020; Kudrin et al., 2021). In addition, the effects of UR on soil nematode communities were stronger than that of the overstory removal. The primary reason may be that the understory vegetation has more important roles in maintain soil micro-climate (e.g., soil temperature and moisture). In the same study site and during the same experimental period, UR significantly increased monthly mean soil temperature, decreased soil water content and decreased litter decomposition rate; and the effects of UR were stronger than the overstory removal (Zhao et al., 2012). In addition, the *Dicranopteris*-dominated understory worldwide is considered as an ecosystem driver having abilities in maintain soil micro-climate (Yang et al., 2021).

In contrast to the latter studies, the current study was conducted in a subtropical region, where high temperatures and high precipitation could result in the rapid decomposition of plant residues and therefore a rapid but temporary increase in nematode abundance in response to vegetation removal. In addition to being explained by a reduction in food resources, the subsequent decline in nematode abundance in response to plant removal could be explained by an increase in soil temperature and a decrease in soil moisture (Zhao et al., 2012).

Our PRC analysis revealed that the effects of plant removal on the temporal dynamics of the soil nematode genera differed in the upper vs. lower soil layer. In the lower soil layer and relative to the control, plant removal had mild effects on nematode abundances during the first four sampling events, but caused sharp decreases of most nematode genera in the fifth sampling event and sharp increases of most nematode genera in the sixth sampling event. This finding was partially inconsistent with our first hypothesis, which stated in part that vegetation removal would have similar effects on nematodes in both soil layers. For the temporal patterns of nematode communities at the 5–10 cm depth at the first 258 days (T1–T5), the mostly likely reason is that the abundance of soil biota is lower at the lower soil depth (Supplementary Table 1) and their requirement for food resources is lower too. The plant roots may maintain the soil nematode communities for a relative long time until resource depletion (T1–T5). However, the sharp increase of nematodes at the last sampling even is difficult to explain. A likely reason is that the trophic cascade in soil food-webs may induce this temporal pattern (Pace et al., 1999; Dyer and Letourneau, 2003). Particularly, relative increases in soil microbial biomass (food supply for nematodes) and/or decreases in the abundance of soil predators (feeding on nematodes) may result in increases in nematode abundance at the last sampling event. Further research is needed to determine whether the increases in the abundances of most nematode genera in the last sampling event would continue. As noted above, researchers have reported two general effects of vegetation removal on soil nematode communities, i.e., vegetation removal suppresses soil nematodes in some cases but initially increases and than suppresses soil nematodes in other cases (Sohlenius, 1982, 1997; Forge and Simard, 2000; Panesar et al., 2000). Because the time is used as a covariate, any eventual seasonal change shared by all plots is removed by the PRC method (Lepš and Šmilauer, 2003). Therefore, the temporal dynamics of soil nematode communities in the lower soil layer in the vegetation removal plots represent net treatment effects. In other words, differences in nematode communities represented by the visualized curves might not result from seasonal change during this study.

Theoretically, the *r*-selected taxa are resistant to ecosystem disturbances and the *K*-selected taxa are sensitive to ecosystem disturbances (Bongers and Bongers, 1998; Ferris et al., 2001). In the current study, however, many typical *r*-selected nematode genera (i.e., cp1 and cp2 guilds) were not resistant to vegetation removal and not all of the *K*-selected nematode genera (i.e., cp4 and cp5 guilds) were sensitive to vegetation removal. This finding was inconsistent with the idea that nematode genera with high cp values (*K*-selected) would be more sensitive to vegetation removal than those with low cp values (*r*-selected). In addition, the effect of vegetation on the compositions of nematode functional guilds was similar to the effect of vegetation removal on the abundance of soil nematode genera (Figure 2). The responses of some guilds to vegetation removal were inconsistent with their theoretical responses. Consistent with our finding, a previous study found that clear-cutting decreased the abundances of most nematode taxa but increased the abundances of some *K*-selected taxa in a pine forest in central Sweden (Sohlenius, 1997); similarly, clear-cutting decreased the abundance of most nematode genera but increased the abundances of some genera (including both low and high cp genera) in forest soils of southern British Columbia (Forge and Simard, 2001). Although the cp scale has greatly contributed to the use of nematodes as ecological indicators, it has long been recognized that the inferred cp values for some nematode taxa may be incorrect, i.e., may not reflect their actual life history strategies (Korthals et al., 1996; Fiscus and Neher, 2002; Neher et al., 2004). A meta-analysis showed that only a few nematode genera (*Diphtherophora*, *Prismatolaimus*, *Tylenchorhynchus*, *Plectus*, *Cruznema*, *Mesorhabditus*, *Mesodorylaimus*, and *Nygolaimus*) respond consistently, i.e., worldwide, to a specific type of disturbance (e.g., cultivation and inorganic or organic fertilization) (Zhao and Neher, 2013). In our study, *Diphtherophora* and *Prismatolaimus* in the upper soil layer were sensitive to vegetation removal, which was consistent with the finding that these genera are sensitive to cultivation (Zhao and Neher, 2013). Therefore, the genera *Diphtherophora* and *Prismatolaimus* may be useful indicators of anthropogenic disturbances worldwide. The *r*-selected genera *Filenchus*, *Ditylenchus*, *Protorhabditis*, and *Acrobeloides* may be indicators of vegetation removal in rotated plantations in south China. The abundance of *Chrysonema*, an omnivorous genus, was greatly increased by vegetation removal in the current study, which is opposite of what was expected based on its cp value of 5. We therefore suggest that the cp value of *Chrysonema* should be reduced. A cp value of 3 for *Chrysonema* may be appropriate, because a cp3 carnivorous genus (i.e., *Stenonchulus*) was also increased by the vegetation removal in the current study. In addition, the cp values for omnivores and carnivores are generally ≥3 (Bongers and Bongers, 1998; Ferris, 2010).

In summary, the PRC method is an alternative way to analyze repeatedly collected and complex community data. For the analysis and visualization of such data, the PRC method may be more useful than ANOVAs. Using the PRC method, we found that the abundances of soil nematode genera in the upper 0–5 cm soil layer were initially increased but subsequently suppressed by vegetation removal in a eucalyptus plantation in south China. The effect of UR on soil nematode genera was stronger than the effect of TR. The effects of vegetation removal on the temporal dynamics of soil nematode communities differed in the lower vs. the upper soil layer. The results also suggested that *Prismatolaimus* and *Diphtherophora* may be good indicators of the effects of vegetation removal in a subtropical forest. The use of the abundances of soil nematode genera as indicators of the effects of specific kinds of disturbances, however, requires reconsideration because the commonly used cp scale incorrectly predicted the responses of some nematode genera to vegetation removal in the current study.

## Data Availability Statement

The original contributions presented in the study are included in the article/Supplementary Material, further inquiries can be directed to the corresponding author/s.

## Author Contributions

SF and JZ designed the experiments. JZ, DG, and SW performed the experiments. DG carried out data analysis and wrote the manuscript. SF and JZ helped in preparing the manuscript and in interpretation of the analyses during constructive discussions. All authors contributed to the article and approved the submitted version.

## Conflict of Interest

The authors declare that the research was conducted in the absence of any commercial or financial relationships that could be construed as a potential conflict of interest.
